# Cytokinin Confers Brown Planthopper Resistance by Elevating Jasmonic Acid Pathway in Rice

**DOI:** 10.3390/ijms23115946

**Published:** 2022-05-25

**Authors:** Xiao Zhang, Daoming Liu, Dong Gao, Weining Zhao, Huaying Du, Zeyu Qiu, Jie Huang, Peizheng Wen, Yongsheng Wang, Qi Li, Wenhui Wang, Haosen Xu, Jun He, Yuqiang Liu, Jianmin Wan

**Affiliations:** 1 State Key Laboratory for Crop Genetics & Germplasm Enhancement, Jiangsu Provincial Research Center of Plant Gene Editing Engineering, Nanjing Agricultural University, Weigang 1, Nanjing 210095, China; zxss25@163.com (X.Z.); liudm0924@163.com (D.L.); 2019101099@njau.edu.cn (D.G.); 2019101096@njau.edu.cn (W.Z.); 2019101098@njau.edu.cn (H.D.); qzy910525@163.com (Z.Q.); hj6751159@163.com (J.H.); 2017201048@njau.edu.cn (P.W.); 2017101098@njau.edu.cn (Y.W.); 2019201069@njau.edu.cn (Q.L.); zzyzwwh@163.com (W.W.); xuhaosen@126.com (H.X.); hj@njau.edu.cn (J.H.); 2 National Key Facility for Crop Gene Resources and Genetic Improvement, Institute of Crop Science, Chinese Academy of Agricultural Sciences, Beijing 100081, China

**Keywords:** cytokinins (CKs), jasmonic acid (JA), rice (*Oryza sativa*), brown planthopper (BPH)

## Abstract

Plants have evolved a sophisticated defense system that employs various hormone pathways to defend against attacks by insect pests. Cytokinin (CK) plays an important role in plant growth and stress tolerance, but the role of CKs in plant–insect interaction remains largely unclear. Here, we report that CKs act as a positive regulator in rice resistance against brown planthopper (BPH), a devastating insect pest of rice. We found that BPH feeding promotes CK biosynthesis and signaling in rice. Exogenous application of CKs significantly increased the rice resistance to BPH. Increasing endogenous CKs by knocking out *cytokinin oxidase*/*dehydrogenase* (*OsCKXs*) led to enhanced resistance to BPH. Moreover, the levels of the plant hormone jasmonic acid (JA) and the expression of JA-responsive genes were elevated by CK treatment and in *OsCKXs* knockout plants. Furthermore, JA-deficient mutant *og1* was more susceptible to BPH, and CK-induced BPH resistance was suppressed in *og1*. These results indicate that CK-mediated BPH resistance is JA-dependent. Our findings provide the direct evidence for the novel role of CK in promoting insect resistance, and demonstrate that CK-induced insect resistance is JA-dependent. These results provide important guidance for effective pest management strategies in the future.

## 1. Introduction

Rice (*Oryza sativa L.*) is among the most important staple foods worldwide [1,2]. Rice is constantly subject to diverse biotic and abiotic stresses. Among the biotic stresses, brown planthopper (BPH; *Nilaparvata lugens* Stål, Hemiptera, Delphacidae) is one of the most destructive insect pests for rice, causing billions of dollars in losses annually. In addition to the direct damage caused by feeding on rice phloem sap, BPH also causes indirect damage by transmitting rice grassy stunt virus and ragged stunt virus [3,4]. Currently, chemical control is the predominant strategy for BPH management, which is costly and harmful to environment [5]. Alternatively, the breeding and cultivation of resistant rice cultivars is more effective and environmentally friendly. In the past few decades, over 40 BPH resistance genes have been identified in rice, 11 of which have been successfully cloned [6,7,8,9]. Nonetheless, how these genes confer effective BPH resistance remain elusive, as reports on the mechanism underlying BPH resistance have been scarce.

Plant defense responses to insects rely on a complex network of hormone signaling pathways. Jasmonic acid (JA) and salicylic acid (SA) are critical signaling components in the regulation of induced defenses against microbial pathogens and insect herbivores. The SA pathway contributes to immune responses against biotrophic pathogens and piercing–sucking insects, while the JA pathway activates defense responses against necrotrophic pathogens and chewing herbivores [10,11]. SA-deficient plants ectopically expressing the bacterial salicylate hydroxylase gene *NahG* or silenced for *OsPAL*s are more susceptible to BPH attack, while overexpressing *OsPAL8*, which increases the level of SA, enhances the resistance against BPH [12,13]. Moreover, the SA pathway participates in *Bph14-*, *BPH29-*, *Bph9-*, and *Bph6-*mediated resistance to BPH in rice [6,14,15,16]. However, the role of JA in BPH resistance remains controversial. A previous study reported that silencing *OsLOX9*/*OsHI-LOX* (*9-lipoxygenase*, a JA biosynthetic gene) enhances BPH resistance, which suggests that JA may negatively regulate BPH resistance [17]. However, silencing *CORONATINE IN-SENSTIVE 1* (*OsCOI1*, a receptor for JA) had no impact on rice resistance against BPH [18]. In addition, a recent study reported that knocking out *AOC* (*Allene Oxide Cyclase*, a JA biosynthetic gene) and *MYC2* (a key transcription factor in the JA pathway) in rice led to susceptibility to BPH [19]. Consistently, exogenous application of JA was also found to significantly increase BPH resistance [16]. These studies support a positive role of JA in BPH resistance, which adds to the complexity of the issue. In view of these controversial results, the role of the JA pathway in BPH resistance needs further investigation.

The key plant hormone cytokinin (CK) is involved in diverse processes of plant growth and development [20,21,22,23,24,25]. The homeostasis of CKs in plants relies on the balance between biosynthetic enzyme adenosine phosphate isopentenyltransferase (IPT) and catabolic enzyme cytokinin oxidase/dehydrogenase (CKX) [20]. Two types of CK response regulators (RRs) play central roles in CK signaling. Type-A RRs usually serve as negative regulators for CK signaling, while type-B RRs are the positive regulators that oppose the negative regulation imposed by type-A RRs [26,27]. In addition to regulating plant growth and development, an increasing number of studies are unveiling the role of CK in various biotic and abiotic stresses [28,29,30,31]. In early studies, CKs were widely recognized to be utilized by gall or green-island-inducing pathogens to manipulate their plant host [32,33]. However, recent findings have shed new light on the positive role of CK in plant immune responses. For instance, elevated levels of endogenous CKs by overexpressing *IPTs* increased the resistance to *Pseudomonas syringae* in *Arabdopsis* [29] and insects in *Nicotiana* [34]. In turn, BPH feeding significantly influenced the expression of CK biosynthesis-related genes and increased the content of CKs [16,35]. However, direct genetic evidence supporting the positive regulation of CKs on BPH resistance is absent. Moreover, the underlying mechanism of CK-mediated BPH resistance is still largely unclear.

Our study showed that BPH feeding leads to increased CK levels in rice by simultaneously upregulating the expression of CK biosynthetic genes and downregulating the expression of CK oxidase/dehydrogenase genes. In addition, either exogenous application of CKs or increasing endogenous CK levels by knocking out CKXs enhanced BPH resistance in rice. In addition, the expression of JA pathway-related genes and the JA content were both upregulated by CK treatment or in CKXs knockout mutants. The CK-triggered BPH resistance was blocked in JA-deficient mutant og1. Together, we demonstrate that CK positively regulates BPH resistance by promoting the JA pathway.

## 2. Results

### 2.1. BPH Infestation Promotes CK Pathway in Rice

**Figure 1:**
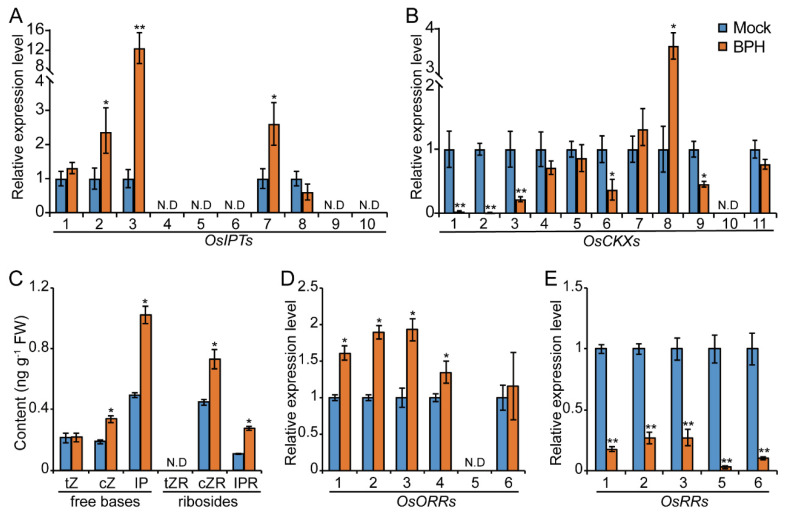
BPH infestation activates the CK pathway in rice. (**A**,**B**) qRT-PCR analysis of transcript levels of CK metabolic genes *OsIPTs* and *OsCKXs* in BPH-infested and non-infested rice seedlings. The expression levels of genes in non-infested rice seedlings were set as 1. *Ubiquitin (Os03g0234350)* was used as an internal reference. (**C**) Levels of endogenous CKs in BPH-infested and non-infested rice seedlings measured by nano-LC-ESI-Q-TOF-MS. tZ, trans-zeatin; cZ, cis-zeatin; iP, isopentenyladenine; tZR, cis-zeatin riboside; cZR, cis-zeatin riboside; iPR, isopentenyladenine riboside. (**D**,**E**) Transcript levels of type-B CK response regulators, *OsORRs*, and type-A CK response regulators, *OsRRs*. Sheaths from ten-day-old seedlings of Zhonghua11 were collected at 24 h after BPH feeding for qRT-PCR analysis and CK quantification. N.D indicates undetected. Data are shown as mean ± SD, *n* = 3. Statistical differences were determined by two-tailed Student’s *t*-test, * *p* < 0.05, ** *p* < 0.01.

To investigate the role of CK in rice resistance against BPH, we analyzed the effect of BPH infestation on the transcript levels of CK metabolic genes and CK signaling genes by qRT-PCR assay. Adenosine phosphate isopentenyltransferases (OsIPTs) are key enzymes for CK biosynthesis [20]. We found that several *OsIPTs*, including *OsIPT2*, *OsIPT3,* and *OsIPT7,* were induced by BPH feeding (Figure 1A). Meanwhile, the expression of *cytokinin oxidase/dehydrogenases* (*CKXs*), which irreversibly inactivate CKs, were downregulated by BPH feeding (Figure 1B). These results indicated that BPH infestation may promote the accumulation of CKs. To test this hypothesis, we compared the concentrations of CKs in sheaths of rice seedlings with or without BPH infestation. Consistent with our hypothesis, both cZ-type (cZR, cZR) and iP-type (iP, iPR) CKs were significantly increased in BPH-infested plants compared with non-infested plants.

Subsequently, we analyzed the expression of genes involved in CK signaling. Several type-B response-regulator-encoding genes, *OsORR1*, *OsORR2*, *OsORR3*, and *OsORR4*, which function as positive regulators in the CK signaling pathway, were induced by BPH infestation (Figure 1C). On the contrary, several type-A response regulators that negatively regulate CK signaling transduction, including *OsRR1*, *OsRR2*, *OsRR3*, *OsRR5,* and *OsRR6* [36,37,38], were suppressed by BPH infestation (Figure 1D). In summary, BPH infestation increases CK content and upregulates the CK signaling pathway.

### 2.2. Exogenous CK Treatment Promotes Resistance to BPH in Rice

**Figure 2:**
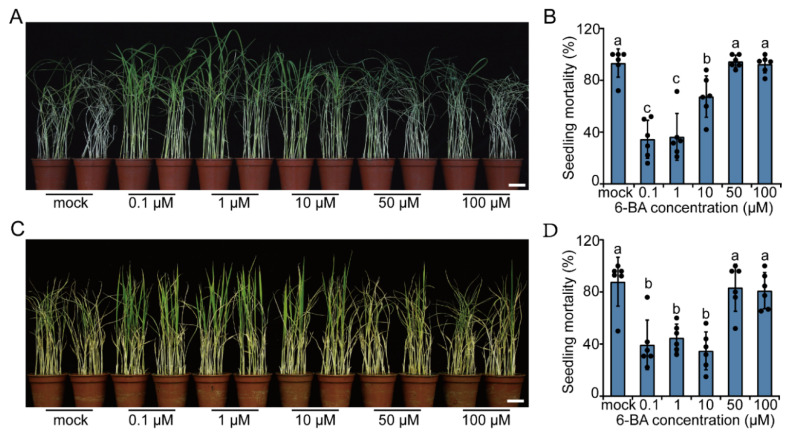
Exogenous CK treatment promotes rice resistance to BPH. Representative images of *japonica* rice cultivar Zhonghua11 (**A**) and *indica* rice cultivar 93-11 (**C**) infested with BPH after pretreatments with mock, 0.1 µM, 1 µM, 10 µM, 50 µM, or 100 µM 6-benzylaminopurine (6-BA). (Scale bars, 3 cm). Mortality rate of *japonica* rice cultivar Zhonghua11 (**B**) and *indica* rice cultivar 93-11 (**D**) seedlings infested with BPH after pretreatments with different concentrations of 6-BA. The mortality of rice seedlings was recorded at day seven post infestation with BPH. For B and D, a dot represents a biological replicate. Data are means ± SD, *n* = 6. Different letters on the columns indicate significant differences at *p* < 0.05 determined by one-way ANOVA with Tukey’s test.

Based on the observation that BPH attack elevates the CK pathway in rice, we wondered whether the CK pathway participates in BPH resistance. Then, we tested the influence of exogenous CKs, using 6-benzylaminopurine (6-BA) (from 0 µM to 100 µM) treatment on rice resistance to BPH. Results showed that exogenously spraying rice seedlings with 6-BA at relatively low concentrations (0.1~10 µM) significantly enhanced rice resistance to BPH, especially at 0.1 µM and 1 µM; however, exogenous treatment with high concentrations of 6-BA (50~100 µM) had no obvious effects on rice responses to BPH infestation (Figure 2A,B). We also determined the influence of exogenous CKs on BPH resistance in *indica* rice cultivar 93-11. Similarly, low concentrations of exogenous 6-BA increased the BPH resistance of *indica* rice cultivar 93-11 (Figure 2C,D), while treatment with high-concentration CKs exerted no influence on BPH resistance. These results demonstrate that CKs at relatively low concentrations promote BPH resistance in a wide range of rice cultivars.

### 2.3. Knocking out OsCKXs Promotes the Resistance to BPH in Rice

**Figure 3:**
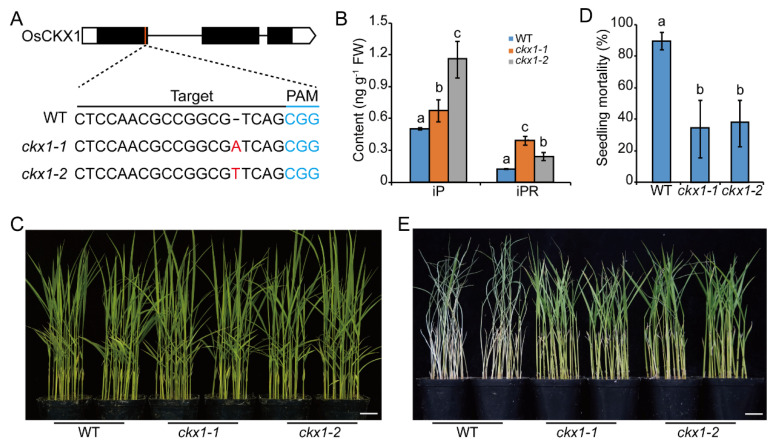
Increasing endogenous CKs level significantly enhances rice resistance against BPH. (**A**) A diagram of the molecular lesion in the *ckx1* mutants generated by CRISPR/cas9 genome-editing technology. The protospacer adjacent motifs (PAM) sequence and guide RNA targeting sites are indicated by overlined and blue color, respectively. (**B**) Contents of iP and iPR in rice sheaths of WT (Kitaake) and *ckx1* seedlings. (**C**) Representative image of WT and *ckx1* mutant seedlings before BPH infestation. Seedling mortality rate (**D**) and representative image (**E**) of WT and *ckx1* mutant at day seven post infestation with BPH. Scale bars, 3 cm. Values are mean ± SD, *n* = 3. Different lower-case letters on the columns indicate significant differences at *p* < 0.05 determined by one-way ANOVA with Tukey’s test.

To further verify the positive role of CKs in BPH resistance, we generated knockout mutants of cytokinin oxidase/dehydrogenases gene *OsCKX1* using a CRISPR/Cas9 genome-editing approach. Two T2 homozygous positive lines were selected for further study (hereafter, *ckx1-1* and *ckx1-2*) (Figure 3A). CKs were significantly increased in both *ckx1* knock-out mutants compared with wild type (WT) (Figure 3B), which was consistent with the known function of *OsCKXs* in CK catabolism. No visible difference was observed between *ckx1* and WT seedlings before BPH attack (Figure 3C). We evaluated the two *ckx1* mutants for resistance level to BPH. While the seedling mortality of WT arrived at almost 90%, *OsCKX1* knockout lines were just about 30% (Figure 3D,E). These results indicate that increasing endogenous CK content can significantly enhance BPH resistance of rice. To confirm this, we collected previously reported *OsCKX*s knockout mutants, including *ckx3*, *ckx5*, *ckx8*, *ckx9*, and *ckx11* [39], and assessed their BPH resistance levels. Similar to *ckx1*, all tested *ckx* mutants displayed elevated resistance to BPH compared with WT (Figure S1). Collectively with the results of exogenous CKs treatment, we have demonstrated that CKs positively regulate the resistance against BPH in rice.

### 2.4. CKs Promote the JA Rather than the SA Pathway in Response to BPH in Rice

**Figure 4:**
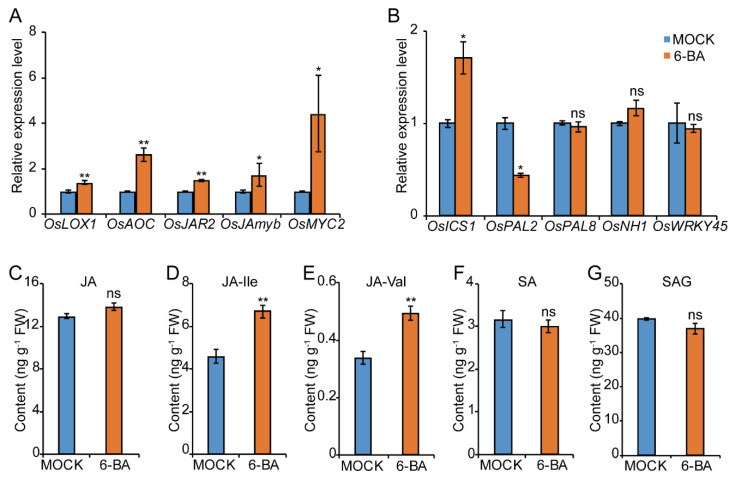
CK promotes the JA pathway in response to BPH in rice. (**A**) Transcript levels of JA-pathway-related genes in rice sheaths pretreated with 0.1 µM 6-BA or mock treatment post infestation with BPH for 24 h, respectively. The expression levels of mock-treated plants were set as 1. *Ubiquitin* was used as an internal reference. (**C**–**E**) Contents of JA and conjugates of JA in rice sheaths pretreated with 0.1 µM 6-BA or mock treatment, respectively, and infested with BPH for 24 h. JA-Ile, Jasmonoyl-L-isoleucine; JA-Val, N-[(-)-Jasmonoyl]-(L)-valine. (**B**) Transcript levels of SA-pathway-related genes in rice sheaths pretreated with 6-BA or mock treatment at 24 h post infestation with BPH, respectively. (**F**,**G**) Contents of SA and salicylic acid 2-O-*β*-Glucoside (SAG) in rice sheaths pretreated with 6-BA or mock treatment at 24 h post infestation with BPH, respectively. FW, fresh weight. Values are means ± SD, *n* = 3. Statistical significances were determined by two-tailed Student’s *t*-test, * *p* < 0.05, ** *p* < 0.01, ns, no significant difference.

Given the important roles of JA and SA in rice–BPH interactions, we investigated whether the CK pathway has crosstalk with the JA or the SA pathway in response to BPH infestation. We analyzed the influence of CKs treatment on transcript levels of JA biosynthesis- and signaling-related genes upon BPH infestation by qRT-PCR assay. Results showed that JA biosynthetic genes *OsLOX1*, *OsAOC*, and *OsJAR2* were significantly induced by spray application of CK (Figure 4A). In agreement with the results of expression analysis, the levels of JA conjugates Jasmonoyl-L-isoleucine (JA-Ile) and N-[(-)-Jasmonoyl]-(L)-valine (JA-Val) were also significantly raised by CK treatment (Figure 4C–E). In addition, application of exogenous CK remarkably upregulated the expression levels of JA signaling-related genes *OsJAmyb* and *OsMYC2* (Figure 4A). Consistently, the transcript levels of JA biosynthesis and signal-related genes were also significantly upregulated, while the JA-Ile and JA-Val contents were also significantly increased in *ckx1* mutants compared with WT (Figure S2). All this points out the positive regulation effect of CK on the JA pathway, which was recently reported in plant–pathogen interactions in *Arabidopsis* [40].

However, except *OsICS1*, the induced expression levels of other SA biosynthetic genes and signal related genes were not observed in exogenous CK treatment (Figure 4B). In addition, though the expression level of *OsICS1* was increased in exogenous CK treatment, SA and SAG (salicylic acid 2-O-β-Glucoside) contents showed no significant differences between exogenous CKs treatment and control, potentially due to the decreased expression of *OsPAL2* (Figure 4B,F,G). These results demonstrate that CK should promote the JA rather than the SA pathway in response to BPH infestation.

### 2.5. CK-Mediated BPH Resistance Depends on the JA Pathway

**Figure 5:**
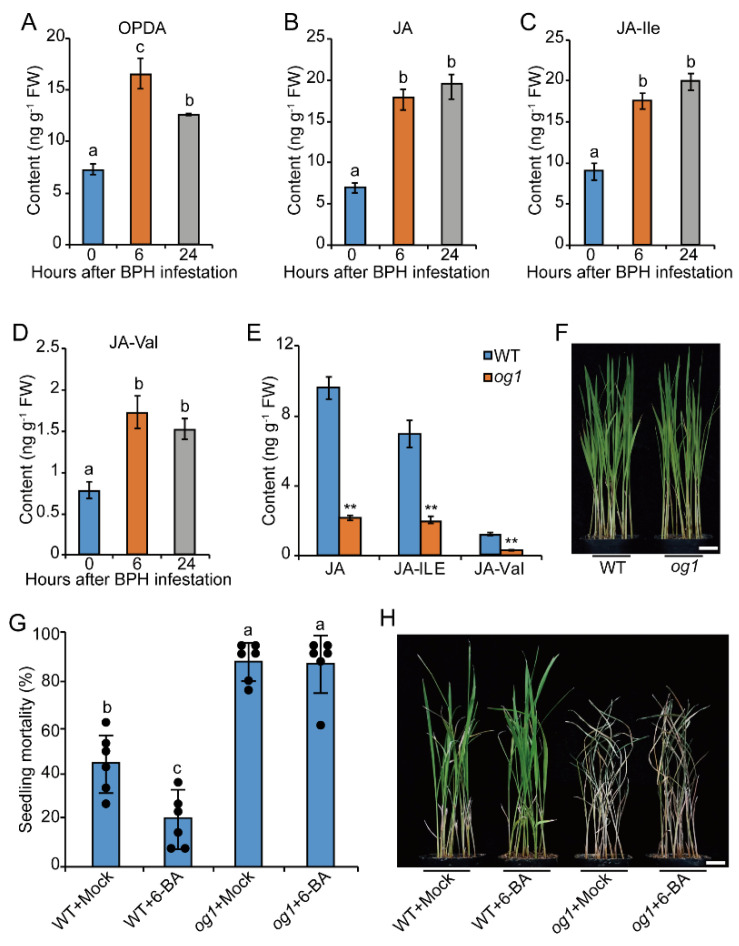
CK positively regulates rice resistance to BPH through the JA pathway. (**A**–**D**) The contents of OPDA, JA, JA-Ile, and JA-Val in sheaths of ten-day-old rice seedlings at 0, 6, and 24 h after infestation with BPH, respectively. (**E**) JA, JA-Ile, and JA-Val contents in *og1* and WT. FW, fresh weight. Values are means ± SD, *n* = 3. Statistical significances were determined by two-tailed Student’s *t*-test, ** *p* < 0.01. (**F**) Representative image of WT and *og1* mutant seedlings before attack with BPH. (**G**,**H**) Seedling mortality and representative image of WT and *og1* pretreated with mock or 0.1 µM 6-BA, then infested with BPH for five days. Data are means ± SD, *n* = 6. A dot represents a biological replicate For (**A**–**D**,**G**), different letters on the columns indicate significant differences at *p* < 0.05 determined by one-way ANOVA with Tukey’s test. Scale bars, 3 cm.

Due to the positive regulation of CK on the JA pathway, we tested the role of JA in BPH resistance. The contents of JA precursor 12-oxo-phytodienoic acid (OPDA), JA, JA-Ile, and JA-Val were all significantly increased by BPH attack (Figure 5A–D), indicating their potential role in BPH resistance. *og1* is a JA-deficient mutant with a loss-of-function mutation in OPDA reductase gene *OsOPR*7, a key JA biosynthesis-related gene [41]. Compared with WT, JA, JA-ILE, and JA-Val contents were significantly reduced in *og1* (Figure 5E). The expression of *OsOPR*7 was significantly induced by BPH attack and 6-BA spray treatment (Figure S3). No significant difference was observed between *og1* and WT at the seedling stage, but after infestation with BPH for five days, the seedling mortality of WT was just about 40%, whereas that of *og1* had arrived at over 90% (Figure 5G,H). These results showed that *og1* displays higher susceptibility to BPH than WT 93-11, which coincides with previous studies that found that JA plays a positive role in BPH resistance [16,19].

To investigate whether CK-mediated BPH resistance is associated with the JA pathway, ten-day-old WT and *og1* seedlings were pretreated with 0.1 µM 6-BA or mock buffer, and then infested with BPH. As shown in Figure 5G,H, exogenous application of 6-BA significantly enhanced the resistance level of WT to BPH, but this disappeared in *og1*. These results demonstrate that CK improves rice resistance to BPH through the JA pathway.

## 3. Discussion

Microbe-derived CKs have long been considered to benefit invaders through hijacking host plant [33,42]. Recent studies have provided increasing evidence indicating that plant-originated CKs play vital roles in resistance responses to various biotic stresses [29,31], however knowledge about the function of CKs in plants’ defenses against insects is limited. Here, we investigated the role of CK in defense responses to a piercing–sucking insect BPH. We found that BPH feeding significantly upregulates the expression of CK biosynthetic genes and increases the content of CKs. Exogenous application with CKs or increased endogenous CKs due to knockout of several *OsCKXs* significantly enhanced rice resistance against BPH. In agreement with the positive role of CKs in resistance against pathogens and other insects, our studies clarified the positive role of CK in defense responses to herbivore insect BPH.

The effects of phytohormones on the physiology of plants are often dosage dependent. In some cases, the effect of the same plant hormone at different concentrations may be opposite. A study in soybeans demonstrated that exogenous application of cytokinin at low concentrations promotes nodule development, while applying high concentrations of cytokinin inhibits the process [43]. As another example, low endogenous JA level promotes, while high JA level inhibits callus formation in plants under in vitro conditions. [44]. Additionally, it is well known that auxin stimulates plant growth at low concentrations but inhibits the process when at higher concentrations. Furthermore, a recent study found that high concentrations of auxin enhances abscisic acid (ABA) responses in *Arabidopsis thaliana*, and this effect is different from that of lower concentrations of auxin [45]. In the current study, we found that the effect of CKs on rice BPH-resistance is also dosage dependent: low concentrations of 6-BA (0.1~10 µM) enhanced the resistance to BPH in rice, whereas high concentrations (50~100 µM) had no significant influence (Figure 2). We speculate that excessive CK may be toxic to rice, weakening the plants. Alternatively, high concentrations of CKs may suppress BPH resistance through cross-talking with other signaling pathways. The molecular mechanism underlying the dosage effect of CKs on BPH resistance needs to be further investigated in the future.

Two types of CK response regulators, type-A RRs and type-B RRs, play central roles in CK signaling. Type-B RRs play pivotal roles in the early responses of plants to CKs, while type-A RRs negatively regulate CK signaling [26,27]. Under normal conditions, the expression levels of type-A *RRs* are significantly induced by CKs [22,36]. By contrast, *B. cinereal* infection of *Arabidopsis* significantly increases the levels of CKs, but the transcript levels of some type-A *ARRs* are inhibited [40,46]. Similarly, we also found that the level of CKs significantly increased upon BPH feeding, but the transcript levels of type-A *RR*s decreased. This is consistent with a previous report that type-A RRs negatively regulate plant immunity in *Arabidopsis* [46]. Based on the results from our study and the previous reports, we reason that rice may promote CK-mediated BPH resistance in two ways. Firstly, BPH feeding may increase the level of CKs by upregulating the expression of CK biosynthesis-related genes, *OsIPTs,* and downregulating *cytokinin oxidase/dehydrogenases* (*CKXs*) (Figure 1). Secondly, BPH feeding may suppress the transcript levels of type-A *RR*s to further enhance CK signaling. However, the molecular mechanism underlying how BPH infestation suppresses the expression of type-A *RRs* is unclear, which is worth further studies.

CKs, as an important category of phytohormones, have been well elucidated in plant growth and development by stimulating cell division to regulate the differentiation and size of the meristems [22,23]. Previous studies showed that CK is implicated in many aspects affecting crop yield, particularly grain number and size. For example, reduced expression of *Gn1a* (*OsCKX2*) in rice causes CK accumulation in inflorescence meristems and increases the number of reproductive organs, resulting in an increase of grain yield [47]. Silencing *CKX1* in barley significantly elevates the plant productivity due to the increase in the number of grains [48]. In addition, increased CKs contribute to tissue repair by stimulating cell division and delay plant senescence [49,50]. CKs also influence plant nutrient translocation by converting source tissues into an active sink and increasing levels of photosynthesis in host leaves [51,52]. Here, we found that knocking out several *OsCKXs* significantly enhances rice resistance to BPH. In light of the dual role of CK in regulating plant defenses against biotic stress and crop yield, this indicates that CK should be a key regulator of the plant growth-defense trade-off, and manipulating CK pathway may be a potential strategy to achieve the purpose of increasing plant resistance and yield simultaneously.

Though previous studies have shown that CK can trigger wound-inducible gene expression and induce the accumulation of insecticidal compounds [53,54], the molecular mechanisms underlying CK-mediated plant resistance to herbivory insects remain largely unknown. CKs can facilitate transcript levels of genes involved in the biosynthesis of multiple plant secondary metabolites and increase their contents, including flavonoid and anthocyanin [55,56]. It is also reported that CKs can elevate the expression of genes involved in lignin biosynthesis and the accumulation of lignin [57]. In addition, previous studies have proved that increasing the accumulation of lignin can facilitate rice resistance against BPH [13]. Consistently, we also found that the expression levels of lignin biosynthesis-related genes were significantly upregulated in plants pretreated with exogenous CKs and *ckx1* mutants (Figure S4), and more lignin was accumulated in *ckx1* compared with WT (Figure S5). Whether CKs mediate BPH resistance through triggering biosynthesis of insecticidal compounds and lignin or in a compensatory way, the underlying molecular mechanisms need further study in the future.

JA and SA are the principal plant defense hormones [10]. Extensive research suggests that SA acts as a positive regulator in BPH resistance [6,12,13,16]. Though the function of JA in rice resistance to BPH is disputed in different studies, increasing studies demonstrate that JA should also positively regulate BPH resistance [15,16,19]. The conflicting results about the role of JA in BPH resistance may be due to the different background in different studies. Here, we found that JA-deficient mutant *og1* displays increased susceptibility to BPH and proved the positive role of JA in regulating BPH resistance against BPH. Hormone networks finely build a plant defense system in antagonistic or synergistic manners [10,11]. The crosstalk between CK and SA or CK and JA have been described in several studies [29,43,58,59]. Many studies showed that CK-mediated plant defense should depend on a SA-dependent way. CK synergistically interacts with the SA pathway and activates the defense response of *Arabidopsis* and rice against pathogens [29,30]. However, we found that exogenous application of CKs has no significant influence on the SA pathway upon BPH infestation. Despite several studies supporting an antagonistic interaction between JA and CK in modulation of callus growth and xylem development, recent research also revealed a synergistic interaction between CK and JA. For example, applying CKs to *N. attenuate* enhanced MeJA-mediated induction of defense metabolites associated with herbivore attack [60]. Furthermore, exogenous CK treatment enhanced the expression levels of JA-related defense genes, but not SA, resulting in higher resistance to *B. cinerea* in *Arabidopsis* [40]. In present study, we also found that the JA pathway was significantly elevated in either exogenous CK treatment or endogenous CK-excessive plants. Moreover, CK-mediated BPH resistance disappeared in JA deficient mutant *og1*. Our studies support that CK activates BPH resistance by synergistically interacting with the JA pathway. These results indicate that the crosstalk relationships between plant hormones may be diverse among different host species and in response to various invaders.

## 4. Materials and Methods

### 4.1. Plant Materials and Growth Conditions

The *japonica* rice Zhonghua11 and *indica* rice 93-11 were selected for exogenous CK treatment. *OsCKX1* was knockout in the background of a *japonica* variety, Kitaake. *Ckx3*, *ckx5*, *ckx8*, *ckx9,* and *ckx11* mutants were kindly provided by Professor Kewei Zhang (College of Chemistry and Life Sciences, Zhejiang Normal University, Jinhua, Zhejiang, China). *og1* is a mutant of *OsOPR7* in the background of *indica* variety 93-11 [41]. All plants were cultivated in an experimental field at Nanjing under natural long-day conditions.

### 4.2. BPH Maintenance

BPH adults were collected in rice fields in Nanjing, and were reared continuously on a BPH-susceptible variety, Taichung Native 1 (TN1), in a greenhouse without exposure to insecticides at 26–28 °C, 60% relative humidity, and a 16:8 h (light:dark) photoperiod.

### 4.3. Evaluation of Rice Resistance against BPH

A seedling bulk test, as described previously, was used to evaluate the rice resistance against BPH [7]. To ensure that all seedlings were at the same growth stage before BPH infestation, seeds were pregerminated. Approximately 30 seedlings of each tested variety or line were sown in 10 cm diameter plastic pots. About seven days after sowing, seedlings were thinned to 25 plants per pot. Then, seedlings were infested with ten 2nd- to 3rd-instar BPH nymphs per seedling at the second-leaf stage. When the average mortality rate of the most susceptible one reached 90%, the mortality rates of other cultivars and lines were recorded. At least three replicates were used for each cultivar and line.

### 4.4. Hormone Treatments

For spray treatment, 6-benzylaminopurine (6-BA) was dissolved in 0.1 M sodium hydroxide solution and diluted into concentrations required for use in water. Corresponding volumes of sodium hydroxide solutions (without 6-BA) were added into 6-BA solutions at different concentrations to guarantee each 6-BA solution contained the same amount of sodium hydroxide. An aqueous solution with an equivalent amount of sodium hydroxide was used as a control treatment. In the BPH resistance evaluation experiment, seedlings were sprayed with mock, 0.1 µM 6-BA, 1 µM 6-BA, 10 µM 6-BA, 50 µM 6-BA, or 100 µM 6-BA at the second-leaf stage for 12 h, then infested with BPH. The leaf sheaths were sampled at 24 h post infestation with BPH for quantitative RT-PCR (qRT-PCR) analysis and hormones measurement. Every experiment was repeated three times.

### 4.5. Phytohormones Measurements

CK (iP, iPR, tZ, tZR, cZ, and cZR), JA (JA, JA-Ile, and JA-Val), and SA (SA and SAG) contents were detected by MetWare (Wuhan, China) (http://www.metware.cn/, accessed on 23 May 2022) based on the AB Sciex QTRAP 6500 LC-MS/MS platform. Three replicates of each assay were performed. Sheaths collected from second-leaf stage seedlings at different timepoints post infestation with BPH were used for CK and JA measurement. Sheaths of second-leaf stage seedlings treated with 0.1 µM 6-BA or mock treatment were harvested at 24 h post infestation with BPH for SA and JA measurement.

### 4.6. Lignin Analysis

The total content of lignin was measured following the method described previously [61]. Every experiment was repeated three times.

Histochemical staining of lignin by phloroglucinol method for cellular observation was conducted as previously described [62]. In brief, fresh hand-cut samples were acquired from rice leaf sheaths of one-month-old rice seedlings, then fixed, sectioned, and stained by phloroglucinol-HCl (3% (wt/vol) phloroglucinol in ethanol:12 N HCL in a 1:2 ratio). The stained sections were visualized under an Olympus BX51 microscope (Olympus Optical, Tokyo, Japan). At least 20 sections were observed for each of the plant materials.

### 4.7. RNA Extraction and Quantitative Real-Time RT-PCR (qRT-PCR)

The RNA extract and qRT-PCR assay were conducted as previously described [12]. Briefly, total RNA was isolated from rice seedlings using the RNA prep Pure Plant Kit (Tiangen, Beijing, China) according to the manufacturer’s instructions. For qRT-PCR assay, total RNA (1 μg) was reverse transcribed to cDNA using a PrimeScript 1st Strand cDNA Synthesis Kit (TaKaRa, Shiga, Japan). The qRT-PCR assay was performed using SYBR Premix Ex TaqTM kit (TaKaRa, Shiga, Japan) and ABI prism 7500 Real-Time PCR System according to the manufacturer’s instructions (Applied Biosystems, Foster City, CA, USA). Rice *Ubiquitin* (*OsUBQ*) was used as the internal control. The experiment was repeated with at least two biological replicates and three technical replicates. The primer sequences for qRT-PCR assay are listed in Supplementary Information, Table S1.

### 4.8. Plasmid Construction and Plant Transformation

To generate the *ckx1* mutant, a 20 bp gene-specific sequence of *OsCKX1* was synthesized and annealed to form oligo adaptors, then cloned into the CRISPR/Cas9 expression vector pOs-Cas9 [63]. The CRISPR/Cas9 plasmids were transformed into *Agrobacterium tumefaciens* strain *EHA105*, and then transformed into calluses of susceptible variety Kitaake. The positive lines were verified by sequence assay. All primer sequences used are listed in Supplementary Information, Table S1.

### 4.9. Statistical Analysis

All statistical analyses were performed by two-tailed Student’s *t*-test with Microsoft Excel software (Albuquerque, NM, USA) or one-way ANOVA followed by Tukey’s test with IBM Statistical Product Service Solutions (SPSS) software (Armonk, NY, USA).

## 5. Conclusions

In summary, we confirmed the positive role of CK in activating rice resistance against BPH. Furthermore, we demonstrated that CK and JA act synergistically to trigger BPH resistance in rice. Although the molecular modulation mechanisms underlying the interactions between CK and JA need to be further studied, our findings will deepen our knowledge about the insect–host plant interaction.

## Data Availability

The data presented in this study are available on request from the corresponding author.

## Supplementary Materials

The following supporting information can be downloaded at: https://www.mdpi.com/article/10.3390/ijms23115946/s1.
