# Supplementary material for: Cytokinin Confers Brown Planthopper Resistance by Elevating Jasmonic Acid Pathway in Rice
Source: Int J Mol Sci. 2022 May 25;23(11):5946. doi: 10.3390/ijms23115946 (PMC9180265; doi:10.3390/ijms23115946)
Supplement: Supplementary file 1 [file ijms-23-05946-s001.zip › ijms-1662011-supplementary.pdf]

International Journal of *Molecular Sciences*

MDPI

*Supplementary Materials*

# Cytokinin Confers Brown Planthopper Resistance by Elevating Jasmonic Acid Pathway in Rice

**Xiao Zhang [1,†], Daoming Liu [1,†], Dong Gao [1], Weining Zhao [1], Huaying Du [1], Zeyu Qiu [1], Jie Huang [1], Peizheng Wen [1], Yongsheng Wang [1], Qi Li [1], Wenhui Wang [1], Haosen Xu [1], Jun He [1], Yuqiang Liu [1,*] and Jianmin Wan [1,*,2]**

[1] State Key Laboratory for Crop Genetics & Germplasm Enhancement, Jiangsu Provincial Research Center of Plant Gene Editing Engineering, Nanjing Agricultural University, Weigang 1, Nanjing 210095, China; zxss25@163.com (X.Z.); liudm0924@163.com (D.L.); 2019101099@njau.edu.cn (D.G.); 2019101096@njau.edu.cn (W.Z.); 2019101098@njau.edu.cn (H.D.); qzy910525@163.com (Z.Q.); hj6751159@163.com (J.H.); 2017201048@njau.edu.cn (P.W.); 2017101098@njau.edu.cn (Y.W.); 2019201069@njau.edu.cn (Q.L.); zzyzwwh@163.com (W.W.); xuhaosen@126.com (H.X.); hj@njau.edu.cn (J.H.)

[2] National Key Facility for Crop Gene Resources and Genetic Improvement, Institute of Crop Science, Chinese Academy of Agricultural Sciences, Beijing 100081, China

* Correspondence: yql@njau.edu.cn (Y.L.); wanjm@njau.edu.cn or wanjianmin@caas.cn (J.W.)

† These authors contributed equally to this work.

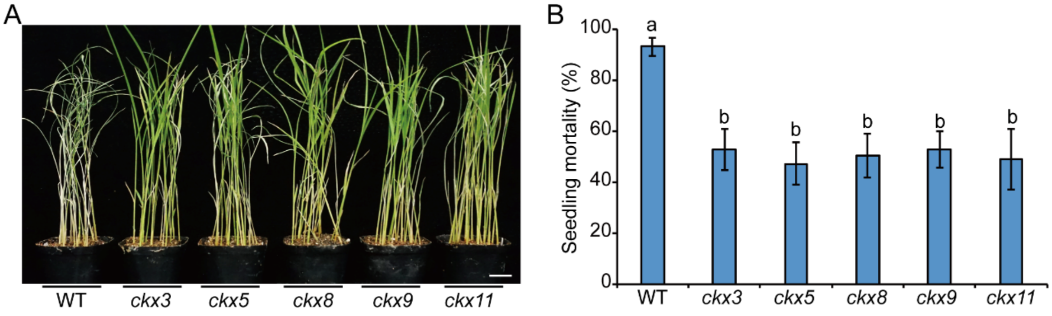


**Figure S1.** Knockout of *OsCKXs* significantly increases rice resistance to BPH. Representative image (A) and seedling mortality (B) of WT and *ckx* mutants. WT (Nipponbare) and *ckx* mutants at second-leaf stage were infested with BPH, and the seedling mortality of each was recorded seven days after BPH infestation. Data are means ± SD, n = 3. Statistical significance was determined by one-way ANOVA with Tukey's test, different letters on the columns indicate significant differences at $P < 0.05$. Scale bar, 3 cm.

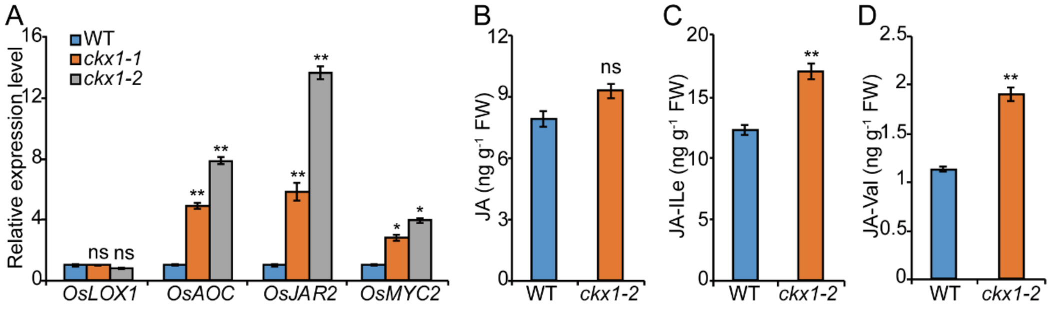


**Figure S2**. Expression analysis of JA pathway related genes and measurement of JA content in *ckx1* mutants. (A) Transcript analysis of JA pathway related genes in *ckx1*. The expression level of WT plant was set as 1. *Ubiquitin* (*Os03g0234350*) was used as an internal parameter. Levels of endogenous JA (B), JA-Ile (C) and JA-Val (D) in *ckx1* and WT. FW, fresh weight. Values are means ± SD, n = 3. Two-tailed Student's *t*-test, *$P < 0.05$, **$P < 0.01$, ns, no significant difference.

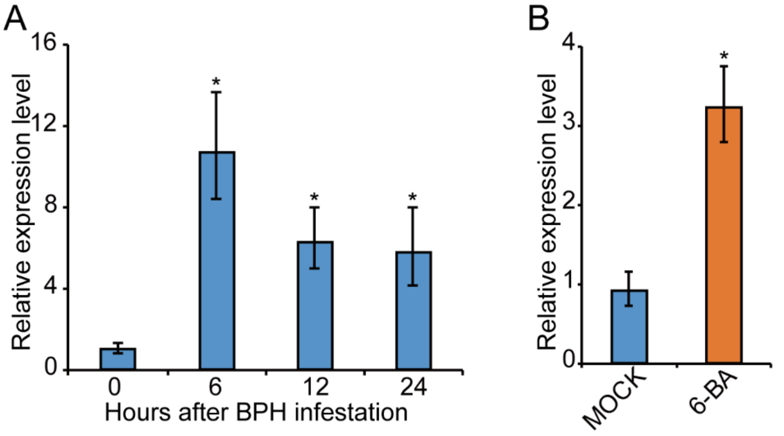


**Figure S3.** Influence of BPH attack and CK treatment on the transcript level of *OsOPR7*. (A) Effect of BPH infestation on the transcript level of *OsOPR7* in rice. The expression level of *OsOPR7* in plants without BPH infestation was set as 1. (B) Expression level of *OsOPR7* in rice seedlings treated with mock and 0.1 μM 6-BA. Results were presented relative to the value of mock-treated plant. *Ubiquitin* (*Os03g0234350*) was used as internal reference. Values are means ± SD, n = 3. Two-tailed Student's *t*-test, **P* < 0.05.

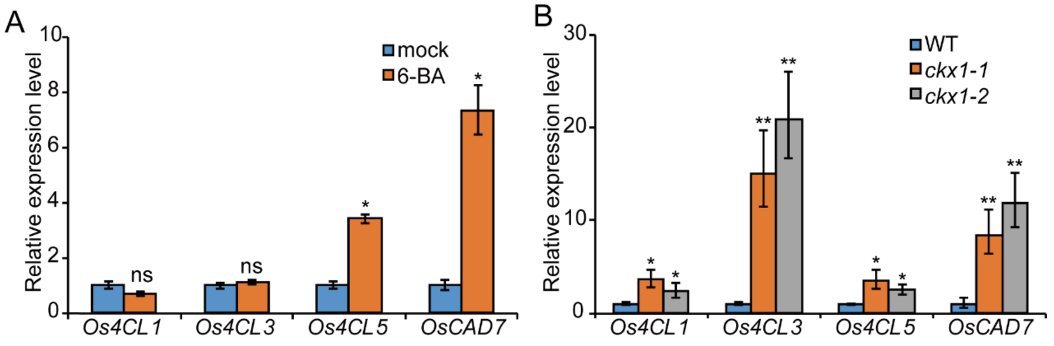


**Figure S4.** CK induces lignin pathway in rice. Transcript levels of lignin biosynthesis-related genes in rice sheaths from plants pretreated with mock or 0.1 μM 6-BA (A) and *ckx1* mutant (B) post infestation with BPH for 24 h. The expression levels of mock-treated plant and WT were set as 1, respectively. *Ubiquitin* (*Os03g0234350*) was used as an internal reference. Two-tailed Student's *t*-test (values are means ± SD, n = 3), **P* < 0.05, ***P* < 0.01, ns, no significant difference.

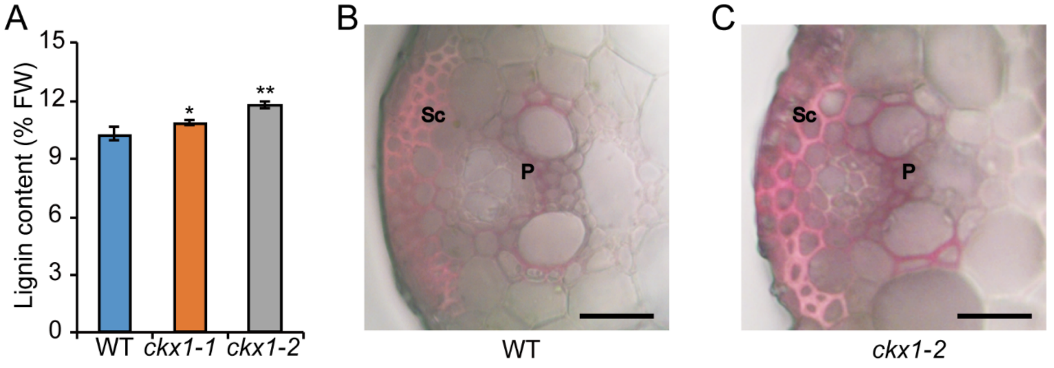


**Figure S5.** CK facilitates the accumulation of lignin in rice sheaths. (A) Quantification of lignin accumulation in fresh leaf sheaths of *ckx1* and WT. FW, fresh weight. Values are mean ± SD of 3 biological replicates. Two-tailed Student's *t*-test, **P* < 0.05, ***P* < 0.01. (B and C) Representative images of lignin accumulation showed by histochemical staining in fresh leaf sheaths of WT and *ckx1*. Sc, sclerenchyma; P, phloem. Scale bar, 30 μm.

**Table S1.** List of primers used in this study.

| **Primers for qRT-PCR - CK metabolism** | | |
|---|---|---|
| **Name** | **Forward Primer** | **Reverse Primer** |
| *OsIPT1* | TCCACCAAGCCCAAGGTTAT | TCGGTGACCTTGTTGGTGAT |
| *OsIPT2* | TCATCGGACAGTCACCCAAG | CACCTGGATCTTGTCGGAGT |
| *OsIPT3* | TCTGCATGGAGGAGGGAATG | CCGCAACCTCCATCTTCTCT |
| *OsIPT4* | GTACGAGTGCTGCTTCCTCTG | CCAGATGCCCCTGGAGTAGT |
| *OsIPT5* | CAGCGTCAGCAGGAGCAT | CGCGGCCGTGAACTCC |
| *OsIPT6* | GATCGATGCGGCATATCTCATC | CCTCCAATTGCCCAAAAGGATC |
| *OsIPT7* | GGATACGAGGATGGTGGTGA | TCGATGGACAGCTTGGTCTT |
| *OsIPT8* | AGACCAAGCTTTCCATCGAC | CAGCTGAATCTTGTCAGCGT |
| *OsIPT9* | TTGTGAGGCCTGTGATAAC | GCTTGTGTTGCTCCCACT |
| *OsIPT10* | GCCATTGCGCAGTTTATC | CACGGAACACCCTGTTCT |
| *OsCKX1* | GGAGGAGGAGGTGTTCTACG | GAGGTCGCAGAACCTCAGTA |
| *OsCKX2* | ACACTGACACACACAAACCG | CAGTTGAGCATGAGGAGCAC |
| *OsCKX3* | GGACAGCATCTCACCGGATA | GTCCCACTTGTGTCTGAGGA |
| *OsCKX4* | TTGAGACCTTCACCGAGGAC | TGAGGATGCCTGTCCTGTTT |
| *OsCKX5* | AAAGGAGAGGTGGTGACCTG | GGTGAACTCGGTGAAGTTGG |
| *OsCKX6* | GTGACCTTCACCAGAGACCA | TGTCCGATTGAGCTGGACTT |
| *OsCKX7* | GGCCACGTTCACTAAAGACC | TCGCTGCTAGAGAAGAAGGG |
| *OsCKX8* | TGAAGCTGGTTTCAGCGAAG | GCTTTGGCACGAACACATTG |
| *OsCKX9* | CCTGGCGTATCACCCTATGT | CTCCTGCATTCGACAGTGTG |
| *OsCKX10* | TCGGACTTTGGCCATATTGT | GTGATAAGCGGATTAGGGCA |
| *OsCKX11* | CGTGGCTCAACCTCTTCATC | CACTTGGACTTGAGCATGGG |
| *OsUBQ* | AACCAGCTGAGGCCCAAGA | CGATTGATTTAACCAGTCCATG |
| **Primers for qRT-PCR - CK signaling** | | |
| *OsORR1* | GCTCACTGGGAGGACTTGAT | CTGCAGCAAGCCGTAAAGAT |
| *OsORR2* | ACCTTCTGCTGCTCTTCCAT | CTTCCAAGCAATGGCCAACT |
| *OsORR3* | GGGACAAGGTTTGGCAACAT | TCCCAGAAAGGATGGTGGAC |
| *OsORR4* | CACTGCAACCTCTTGAGTCG | GGTGGCTTGCGACATGTTAT |
| *OsORR5* | GTGCAGTCAAATGGTGGGTT | CAGTTTGCAAGGTCCGGAAA |
| *OsORR6* | TCAGGACAGGAAGCCATCAG | TAGGTCACAGCGTTCATGGT |
| *OsRR1* | GGCGAAACTGGGCAATAG | GCCTCCACAAGGAGATGATACT |
| *OsRR2* | ATTTTGCTGAGAGAGAAAAGA | ACGACACCAGATGCCCACTC |
| *OsRR3* | CGCAGCTCCAAATATCGAGTT | CACATTCCGATCCAGGCTGAG |
| *OsRR5* | ACTACTGGATGCCGGAGATG | CTCCGAGGACATGATCACCA |
| *OsRR6* | CACAATGTTGAAATGGCACCA | AATTAATGTCAAAACTCTGACC |
| **Primers for qRT-PCR – JA and SA pathways** | | |
| *OsLOX1* | GTACGCTGGGTTCACAGCTC | TCAGATGGATGTGCTGTTGG |
| *OsAOC* | CGTACCTGACCTACGAGGAG | GCCCTTGAGGTAGAAGGTGT |
| *OsJAR2* | AGAAGGTTCTCCGCCACTAC | CGGAGCTGAAGAAGACGTTG |
| *OsJAmyb* | GAGGACCAGAGTGCAAAAGC | CATGGCATCCTTGAACCTCT |
| *OsMYC2* | AGCTCAACCAGCGCTTCTAC | CCTTCTTGAGCGACTCCATC |
| *OsICS1* | TATGGTGCTATCCGCTTCGAT | CGAGAACCGAGCTCTCTTCAA |
| *OsPAL2* | AGCTGGTCAACGAGTTCTACA | GAGGGAGTTGACGTCCTGGTT |
| *OsPAL8* | GTGACGAATGTCGCCAAGAA | ATGAGTGGCAAGCTGGATCT |
| *OsNH1* | TTTCCGATGGAGGCAAGAG | GCTGTCATCCGAGCTAAGTGTT |
| *OsWRKY45* | TTCCTTGTTGATGTGTCGTCTC | CCCCCAGCTCATAATCAAGAAC |
| **Primers for CRISPR** | | |
| CR-CKX1-F | GGCAGCGTCAGCGGGCAGACTTAC | AAACGTAAGTCTGCCCGCTGACGC |
